# A Retrospective Review of the Clinical Significance of the Outcome Questionnaire (OQ) Measure in Patients at a Psychiatric Adult Partial Hospital Program

**DOI:** 10.7759/cureus.13830

**Published:** 2021-03-11

**Authors:** Eduardo D Espiridion, Adeolu O Oladunjoye, Udema Millsaps, Maria Ruiza Yee

**Affiliations:** 1 Psychiatry, Drexel University College of Medicine, Philadelphia, USA; 2 Psychiatry, West Virginia School of Osteopathic Medicine, Lewisburg, USA; 3 Psychiatry, West Virginia University School of Medicine, Martinsburg, USA; 4 Psychiatry, Philadelphia College of Osteopathic Medicine, Philadelphia, USA; 5 Psychiatry, Reading Hospital Tower Health, West Reading, USA; 6 Medical Critical Care, Boston Children's Hospital, Boston, USA

**Keywords:** outcome questionnaire, adult partial hospital program, clinical significance, treatment progress

## Abstract

Introduction

Outcome Questionnaire (OQ) measure is becoming a more popular assessment tool for monitoring treatment progress in psychiatry at different settings including inpatient and outpatient settings. It can also be used in non-clinical populations. However, little is known about the evaluation of this tool in the Adult Partial Hospital Program (PHP).

Methods

We conducted a study among patients in an Adult PHP where we extracted data from the OQ analysis program recorded for patients from January 1, 2015 to July 31st, 2020.

Results

We studied a total of 742 patients among which 509 (68.4%) were males. The mean age was 38.58 ± 14.86 years. Most of the patients had depressive disorder (56.9%). The mean numbers of days on admission were 17.37 ± 25.29 days. There is a consistent decrease in the total score average OQ score from initial to final measure with the year 2019 being 31.99 followed by 2017 (30.05) then 2020 (29.56) then 2015/2016 (28.38) and 2018 (27.27) p < 0.001. Also, for treatment progress it was observed that in years 2015/2016, there was significant improvement in 71.67% of the patients; in 2017, there was significant improvement in 78.53% of the patients; in 2018, there was significant improvement in 77.71% of the patients; while in 2019, there was significant improvement in 76.05% of the patients, and in 2020, there was significant improvement in 70.18% of the patients.

Conclusion

The direct benefit of the OQ measure to patients is to provide objective measurements of assessing clinical improvement or deterioration in the treatment progress of their clinical condition. Our study has proved that this is a useful tool to assess such in the Adult PHP.

## Introduction

In mental health care, apart from access to health care, appropriate treatment and perception of care are very important in the management of patients [1]. The use of outcomes measures through rating scales has been widely popular in the last few decades. Outcome assessment helps in defining treatment goal criteria and monitoring the efficacy of treatments [2]. Research has been ongoing to assess the quality of services and track patient progress when such services are provided. Most measures help in evaluating psychotherapy and assess improvement in patient’s clinical outcomes as they are being followed up. One of these measures is the Outcome Questionnaire (OQ) measure which is becoming a more popular assessment tool ranking seventh most common standardized outcome measurement reported by healthcare providers in recent times [3]. OQ measure has been used in psychiatry in different settings including inpatient and outpatient settings. It can also be used in non-clinical populations [4].

About 30 years ago, pioneers of routine outcomes research, Dr. Michael Lambert and Dr. Gary Burlingame developed the OQ assessment tool. While most mental health questionnaires were designed to address specific symptoms and assign diagnoses, these researchers developed the OQ assessment tool to track changes during patient treatment and provide algorithms for detecting negative outcomes prior to treatment failure. The advantage of this assessment tool is that it is brief and inexpensive [5]. This measure has been used in other outcome management studies that assess the quality of improvement efforts from clinically significant changes [6,7]. OQ measure is designed to assess common symptoms across a wide range of mental disorders and syndromes [8]. It is used among many populations, treatment types and modalities allowing for noninvasive clinical use in most care settings.

However, little is known about the evaluation of this tool in Adult Partial Hospital Program (PHP). Adult PHP is a structured program of outpatient psychiatric services as an alternative to inpatient psychiatric care. They provide day treatment but not an overnight stay. It is usually recommended as a transitional step between inpatient and outpatient programs. Patients in the program are seen at interval visits where they are given treatment such as psychotherapy and assessed from time to time on improvement in their psychiatric conditions.

The purpose of this research is to access the predictive value of the OQ measure by describing the clinical improvement or worsening conditions of mental illness in patients at a psychiatric Adult PHP. To the best of our knowledge, this is the first article to report the assessment of this tool in a psychiatric Adult PHP. Since this transition is a critical part of certain mental health conditions, it is helpful to understand if this is a useful tool in assessing the clinical improvement or worsening conditions of mental illness in patients at the psychiatric Adult PHP.

We sort to assess the predictive value of the OQ tool by describing the clinical improvement or worsening conditions of mental illness in patients at a psychiatric Adult PHP.

## Materials and methods

Setting

This retrospective study was conducted among patients in an Adult PHP at a community hospital. The study was approved by the Reading Hospital Institutional Review Board with IRB reference number IRB 052-20. Investigators used the OQ analysis program to extract OQ outcomes data recorded from questionnaires filled out for patients seen in the Adult PHP from January 1, 2015 to July 31st, 2020.

Measures and procedures

OQ tool is a self-reported scale designed to track and measure progress in patients by assessing common symptoms across a wide range of mental disorders and syndromes [8]. It measures three subscales which help to focus on certain problematic areas of treatment that need to be addressed.

Points are assigned for each response using a 5-point Likert scale. Most questions are on a scale of Never (0), Rarely (1), Sometimes (2), Frequently (3), and Almost Always (4). Some questions are reversed scored, i.e., Never (4), Rarely (3), Sometimes (2), Frequently (1), and Almost Always (0). Individual subscales are totaled using the addition of the questions that relate to that subscale. Higher scores indicate more severe distress and functional impairment. The Symptom Distress subscale contains 25 items, and scores range from 0 to 100. The Interpersonal Relations subscale contains 11 items, and scores range from 0 to 44. The Social Role subscale contains nine items, and scores range from 0 to 36. A total score is calculated by summing the subscales, and scores range from 0 to 180. Nine of the items, measure the presence of positive mental states evenly distributed across the three subscales. The instrument's administration and scoring manual provide thresholds for clinically significant distress and impairment, and for reliable change. The higher the score, the more clinical distress the patient is acknowledging.

This study utilized the total score to assess the clinical significance of the OQ-45.2 assessment tool. If a total score of an individual has a positive change on the OQ-45.2 (i.e., a change of 14 points), that individual is defined to have made clinically significant improvement. However, if an individual makes a negative change of 14 points that means that the individual has made a clinically significant deterioration. We derived an initial and final outcome score for each patient. The initial questionnaire was taken as baseline, while the latest questionnaire was defined as final.

Statistical analysis

All statistical analyses were performed using the SPSS statistics package (SPSS Inc., Chicago, IL, USA). Initially, OQ 45.2 measure conducted among the patients was extracted from the OQ analysis program and transferred to an excel spreadsheet for data collation and analysis. SPSS statistics package was then used for further data analysis of collated data. Categorical variables were reported as numbers and percentages while continuous variables were presented as mean and standard deviation. The level of significance will be set at p < 0.05.

## Results

We studied a total of 742 patients who attended the Adult PHP from 2015 to 2020, among whom were 509 (68.6%) males. There was a relatively even age distribution in each decade classification with the highest proportions in ages 21-30 (24.8%), 31-40 (21.2%) and 41-50 (20.8%). The overall mean age was 38.58 ± 14.86 years. Most of the patients had a mood disorder diagnosis, with the highest being depressive disorder (56.7%) followed by bipolar or related disorder (13.3%) and anxiety disorder (9.8%) (Table 1).

**Table 1:** Baseline characteristics of all patients with OQ data in the Adult Partial Hospital Program ADHD: Attention deficit hyperactivity disorder; OQ: Outcome questionnaire.

Sociodemographic data	Frequency (N)	Percentage (%)
Age (years)	Mean 38.58 (±14.86)	
18-20	83	11.1
21-30	184	24.8
31-40	157	21.2
41-50	154	20.8
51-60	107	14.4
> 60	57	7.7
Sex		
Male	233	31.4
Female	509	68.6
Diagnosis		
ADHD	4	0.5
Adjustment Disorder	8	1.1
Anxiety Disorder	73	9.8
Autistic Disorder	1	0.1
Bipolar or Related Disorder	98	13.3
Borderline Personality Disorder (BPD)	8	1.1
Depressive Disorder	420	56.7
Disruptive Behavior Disorder	3	0.4
Personality Disorder (not BPD)	4	0.5
Psychotic Disorder	4	0.5
Schizophrenic Disorder	10	1.3
Substance-related Disorder	9	1.2
Trauma or Stress-related Disorder	26	3.5
Unknown Diagnosis	74	10.0
Days on Admission	Mean 17.37 (±25.29)	
1-7 days	60	8.1
8 days – 14 days	321	43.3
15 days – 21 days	243	32.7
22 days – 28 days	80	10.8
> 28 days	38	5.1
Year Administered		
2015	33	4.4
2016	147	19.8
2017	163	22.0
2018	175	23.6
2019	167	22.5
2020 (January-July)	57	7.7

The mean number of days on admission in this cohort was 17.37 ± 25.29 days with the highest number of patients on admission between days 8-14 (43.3%). There was a relatively even distribution of the number of patients seen across the years observed with the highest being in 2018 (23.6%) followed by 2019 (22.5%) and 2017 (22.0%) (Table 1).

**Figure 1:**
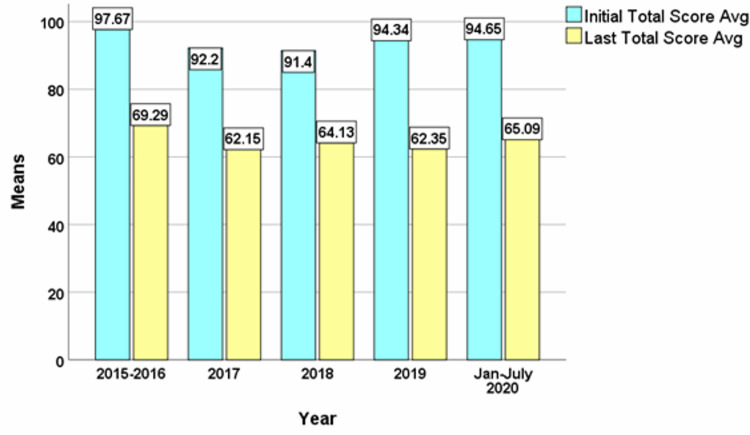
Comparison of the Means of the Adult PHP OQ data OQ: Outcome Questionnaire; PHP: Partial Hospital Program; Avg: Average.

Comparing the means of the OQ scores of the patients in the Adult PHP across the years, there is a consistent decrease in the total score average OQ score from initial to final measure. The largest difference being year 2019 (31.99) followed by 2017 (30.05) then 2020 (29.56) then 2015/2016 (28.38) and 2018 (27.27) p < 0.001 (Figure 1).

**Figure 2:**
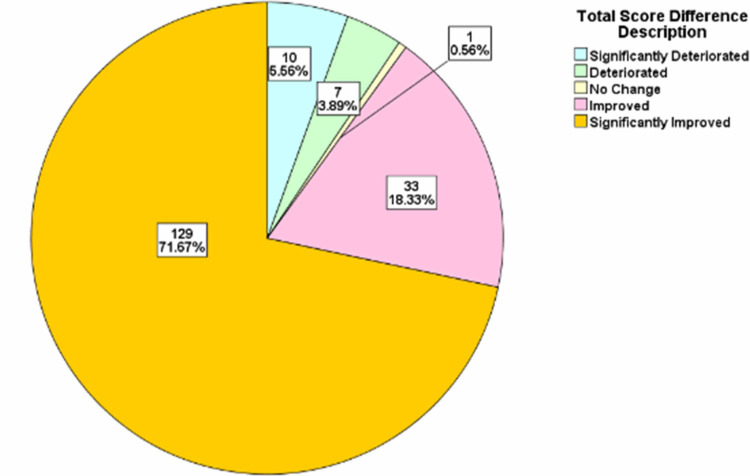
Total score difference description for patients in 2015-2016

**Figure 3:**
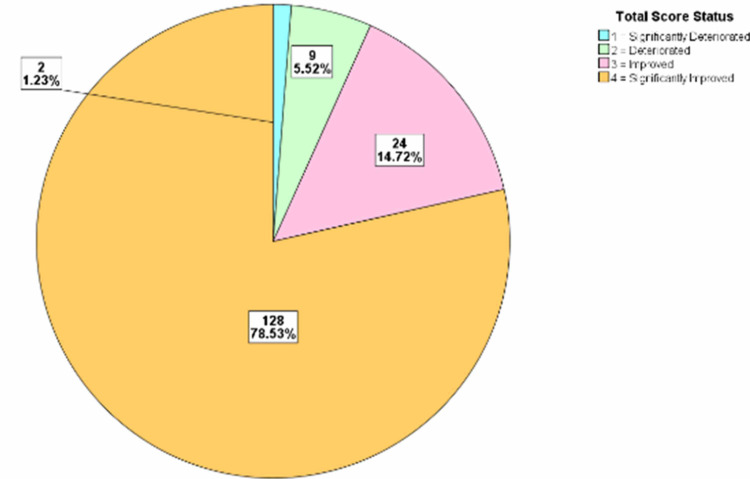
Total score difference description for patients in 2017

**Figure 4:**
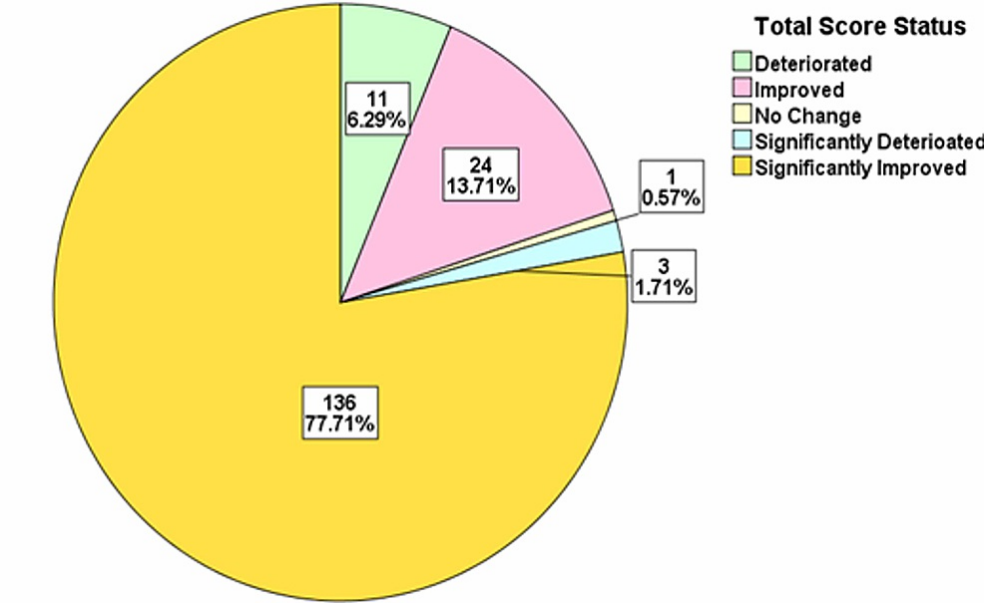
Total score difference description for patients in 2018

**Figure 5:**
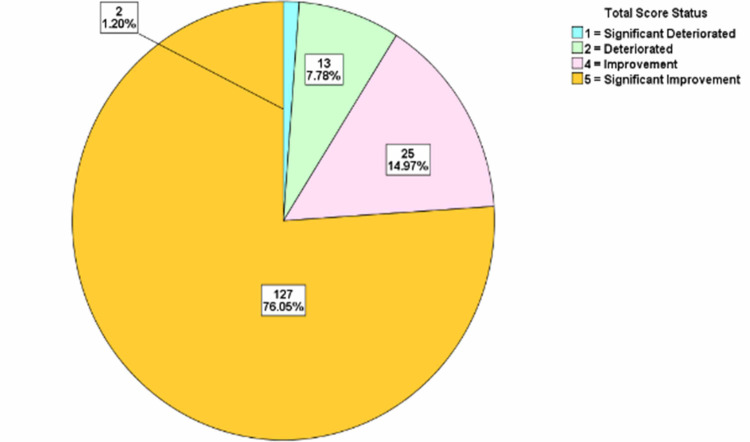
Total score difference description for patients in 2019

**Figure 6:**
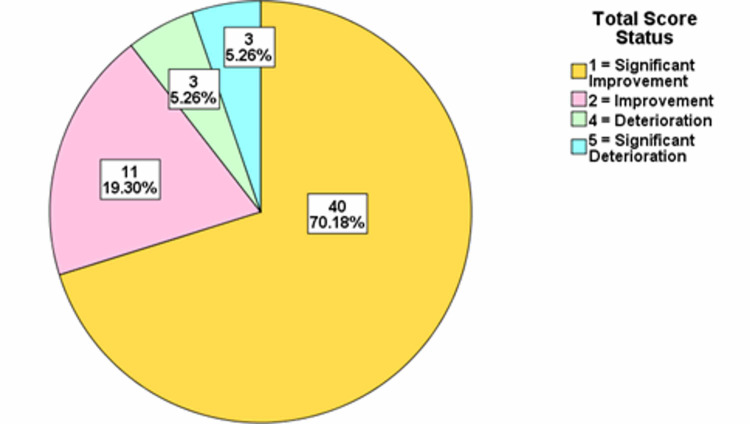
Total score difference description for patients from January-July 2020

For each of the years, the total score difference was assessed. The distributions were classified into significant deterioration, deterioration, no change, improvement, and significant improvement categories. The treatment progress showed that in 2015/2016 there was significant improvement in 71.67% of the patients, while in 2017 there was significant improvement in 78.53% of the patients. In 2018, there was significant improvement in 77.71% of the patients, while in 2019, there was significant improvement in 76.05% of the patients and in 2020, there was significant improvement in 70.18% of the patients (Figures 2-6).

## Discussion

The OQ measure is one of the most frequently used psychotherapy measures in research and clinical setting [3]. The OQ measure is designed to be repeatedly administered enabling the clinician to track and measure the progress of patients as improvement, deterioration, or no change based on a wide range of psychiatric disorders and syndromes [8]. This measure addresses areas vulnerable to changes including, levels of psychiatric symptoms, performance in various roles, interpersonal functioning and levels of quality of life or life satisfaction [8]. We utilized the total score in this study to estimate clinical significance. Clinical significances were classified into five various categories including significant deterioration, deterioration, no change, improvement, and significant improvement. Previous studies have shown categories of clinical outcomes in patients including deterioration (negative change of 14 or more points), no changes (i.e., no improvement or deterioration), improvement (positive change of 14 or more points), and recovered (i.e., shift in scores from dysfunctional to functional range) [9]. In our study, we report that there were a large number of patients with significant improvement across the study period all above 70%. A similar study reported that given cut-off scores, about 32% of their patients met the criteria for clinically significant changes using the OQ measure compared to other available tools giving clinically significant changes ranging from 7% to 23% [8]. This is a remarkable finding in the OQ measure that highlights how effective this tool can be in assessing the clinical improvement of psychiatric patients. This finding suggests that OQ measure producing higher estimates of changes compared to other available tools (such as the Symptom Checklist-90-Revised; the Social Adjustment Rating Scale-Self Report; the Inventory of Interpersonal Problems-Short Form; and the Quality of Life Inventory) might mean that it can be a very useful tool in detecting the effect of even minimal treatment intervention for psychiatric patients such as psychotherapy especially in the setting of an Adult PHP [8].

Also remarkable is the difference in the changes comparing the initial and final total score in our patient population which showed that across the years of study, there was a significant mean difference between the two total scores. The year 2019 had the widest mean difference with 31.99 followed by 2017 with 30.05, then 2020 with 29.56, then 2015/2016 with 28.38 and 2018 with 27.27. The consistency in these findings provides more information on the usefulness of the OQ measure for monitoring treatment progress.

However, the use of OQ has been criticized by some researchers. A study in 2010 by Kim et al. reported different findings from those in the original OQ manual by Lambert et al. when testing the validity of the tool with alternative models [10, 11]. While many researchers continue to affirm its relevance as a strong conceptual model for psychiatric patients, Kim et al. argue that there are still issues with the rationale of some of the OQ items/questions used in determining the scores [10, 12]. On the other hand, many recent studies such as Bludworth et al. that tested several models with the OQ measure in a sample of 1,100 counseling center patients and have found that the OQ measure model fits better for mental health patients than any alternative model, thus strongly endorsing this model [12]. So far also in our study, we did not find any unique characteristics in the PHP patients that would limit the application OQ in the PHP setting. Further research will be needed to continue to test the validity of this model among other groups of patients both in the clinical and non-clinical setting. However, our findings supported by other studies show that the OQ measure has proved its effectiveness in monitoring treatment progress in psychiatric patients, especially in an Adult PHP.

## Conclusions

The direct benefit of the OQ measure to patients is to provide objective measurements of assessing clinical improvement or deterioration in the treatment progress of their clinical condition. Our study has proven that this can be possible in an outpatient setting like the Adult PHP as a few other studies have mentioned in some other clinical settings. More research is needed to establish this model as a vital tool to help both health care providers and patients in assessing treatment progress in psychiatric patients, including in the PHP setting.
